# Correlations of Ion Composition and Power Efficiency in a Reverse Electrodialysis Heat Engine

**DOI:** 10.3390/ijms20235860

**Published:** 2019-11-22

**Authors:** Fabao Luo, Yang Wang, Maolin Sha, Yanxin Wei

**Affiliations:** 1 School of Chemistry and Chemical Engineering, Hefei Normal University, Hefei 230061, China; franksha@aliyun.com (M.S.); yxwei73@mail.ustc.edu.cn (Y.W.); 2 Anhui Province Key Laboratory of Environment-friendly Polymer Materials, Anhui University, Hefei 230601, China; 3 CAS Key Laboratory of Soft Matter Chemistry, Collaborative Innovation Center of Chemistry for Energy Materials, School of Chemistry and Materials Science, University of Science and Technology of China, Hefei 230026, China; youngw@mail.ustc.edu.cn

**Keywords:** salinity gradient power, reverse electrodialysis, concentration difference, electrolyte composition

## Abstract

The main objective of this study is to explore the influence of ion composition on the trans-membrane potential across the ion exchange membrane (IEM), and thus offers a reference for the deep insight of “reverse electrodialysis heat engine” running in the composite systems. In comparison to the natural system (river water | seawater), the performance of the reverse electrodialysis (RED) stack was examined using NaHCO_3_, Na_2_CO_3_, and NH_4_Cl as the supporting electrolyte in the corresponding compartment. The effect of flow rates and the concentration ratio in the high salt concentration compartment (HCC)/low salt concentration compartment (LCC) on energy generation was investigated in terms of the open-circuit voltage (OCV) and power density per membrane area. It was found that the new system (0.49 M NaCl + 0.01 M NaHCO_3_|0.01 M NaHCO_3_) output a relatively stable power density (0.174 W·m^−2^), with the open-circuit voltage 2.95 V under the low flow rate of 0.22 cm/s. Meanwhile, the simulated natural system (0.5 M NaCl|0.01 M NaCl) output the power density 0.168 W·m^−2^, with the open-circuit voltage 2.86 V under the low flow rate of 0.22 cm/s. The findings in this work further confirm the excellent potential of RED for the recovery of salinity gradient energy (SGP) that is reserved in artificially-induced systems (wastewaters).

## 1. Introduction

With the exhaustion of conventional fossil fuels and the excessive emission of greenhouse gas (CO_2_), the demands on renewable energy has grown in the past decades. Salinity gradient energy (SGP) was recognized as one kind of blue energy which was reserved in seawater and river water. It was estimated that 2.4–2.6 TW energy was available by discharging rivers into oceans [1,2], based on Gibbs free energy of mixing. Using reverse electrodialysis (RED) as an energy conversion strategy, it is possible to recover SGP in the natural environment through an economically competitive and environmentally friendly manner. It has attracted growing attention because of its inherent advantages, such as clean and pollution-free, and simple installation [3,4,5]. RED uses ion exchange membranes as the separators, and allows the perm-selective transportation for cations and anions [6,7,8]. When the solution with different concentration is introduced into the corresponding compartment, the ions move across the correlative membranes, and the ion flux is transferred into the electron flux on the electrode. Generally, redox ion pairs (i.e., Fe^3+^/Fe^2+^) were used as the supporting electrolyte in the anode and cathode, or salt/base/acid supporting electrolyte to create water electrolysis circumstance [9,10]. The overall potential could be raised according to the requirements, by repeatedly assembling the membrane pairs (one membrane pair: 1 pc. cation exchange membrane + 1 pc. anion exchange membrane).

The natural system of river water|seawater was the dominant case which has been comprehensively investigated because of its abundant deposits [2,11,12]. The investigations on the optimization of ion exchange membranes or the membrane stack assembly were also the hotspots for the improvement of power density and the SGP recovery efficiency [5,11,12,13,14,15].

Some works reported a novel “reverse electrodialysis heat engine” technique that used ammonium bicarbonate (NH_4_HCO_3_) as the supporting electrolyte, and the waste heat as the “driven-force” to push the reaction NH_4_HCO_3_↔NH_3_+CO_2_+H_2_O forwards. In the discharging circle, NH_3_ and CO_2_ gas dissolved in the concentrated solution, and the free ions (NH_4_^+^, HCO_3_^−^, CO_3_^2−^) diffuse across the ion exchange membrane (IEM) following the classical RED principle. Consequently, the decomposition of NH_4_HCO_3_ is the charging circle which recovers the diffused NH_4_HCO_3_ into the NH_3_ and CO_2_ gas. Recently, it was found the power density of the reverse electrodialysis heat engine system was slightly lower than the natural system on the basis of identical molar concentrations [16,17]. Meanwhile, the solution pH flow cell for converting waste carbon dioxide into electricity was also investigated [18].

Here in this work, we investigated the ion composition on the influence of energy recovery in the discharging circle of the heat engine. By using NaHCO_3_, Na_2_CO_3_, NH_4_Cl, and the composited solutions of NaCl + NaHCO_3_, NaCl + Na_2_CO_3_, NaCl + NH_4_Cl as the supporting electrolyte, we investigated the changes in the trans-membrane potential and power recovery in respect to the ion composition. Then, we provide deep insights into the reliability of the heat engine in complex compositions.

## 2. Results and Discussion

### 2.1. The Influence of Ion Species on Trans-Membrane Voltage

Lots of theoretical models and experimental studies have focused on improving the RED performance by means of salt concentration difference between adjacent ion exchange membrane, according to the Nernst theory [19]. Here in this work, RED was operated by introducing high electromotive force and changing the ion composition in both the high salt concentration compartment (HCC) and low salt concentration compartment (LCC). The system used in the experiments are listed in Table 1.

**Figure 1:**
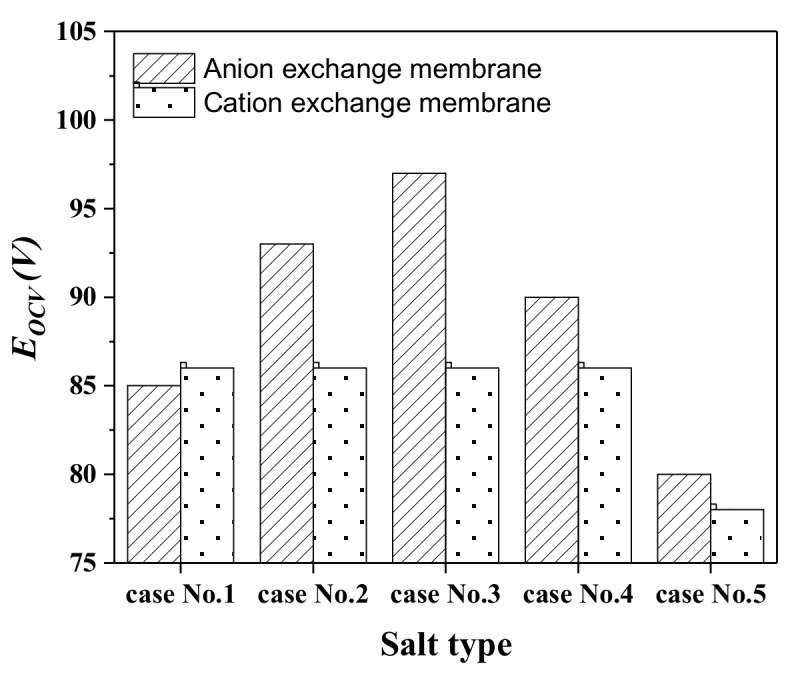
The transmembrane voltages on the AEM and CEM for Case No. 1–5.

The trans-membrane voltage for the anion exchange membrane (AEM) and cation exchange membrane (CEM) was investigated and given in Figure 1. It was found that the trans-membrane voltage across the CEMs and AEMs was around 85 mV in the simulated seawater|river water system (0.5 M NaCl|0.01 M NaCl), which is close to the theoretical Nernst potential. The finding here proves the ion exchange membranes used in the experiment have the desired transport number, and perform perm-selectivity toward the counter-ions well (i.e., CEM allows the transition of cations (counter-ion) and block the anions (co-ion), vice versa for AEM). When the electrolyte in the solution was changed to Na_2_CO_3_, NaHCO_3_, or NH_4_Cl, the changes in transmembrane voltage drop was noticed. However, the changes in AEMs and CEMs are absolutely different. For system Case No.2, the trans-membrane voltage on AEM increases to ca. 93 mV. It was further increased to ca. 97 mV on AEM in the 0.49 M NaCl + 0.01 M NaHCO_3_|0.01 M NaHCO_3_ system, without significant changes in CEM. However, the trans-membrane voltage on AEM decreased to 90 and 80 mV when changing the system to 0.49 M NaCl + 0.005 M Na_2_CO_3_|0.005 M Na_2_CO_3_ and 0.49 M NaCl + 0.01 M NH_4_Cl|0.01 M NH_4_Cl. The trans-membrane voltage changes in CEM was only found in the 0.49 M NaCl + 0.01 M NH_4_Cl|0.01 M NH_4_Cl system (Case, No.5) system, which is about 78 mV. The changes were mainly attributed to ion species transporting across the membrane matrix, and could be calculated according to the Nernst equation for the multi-component case.

**Figure 2:**
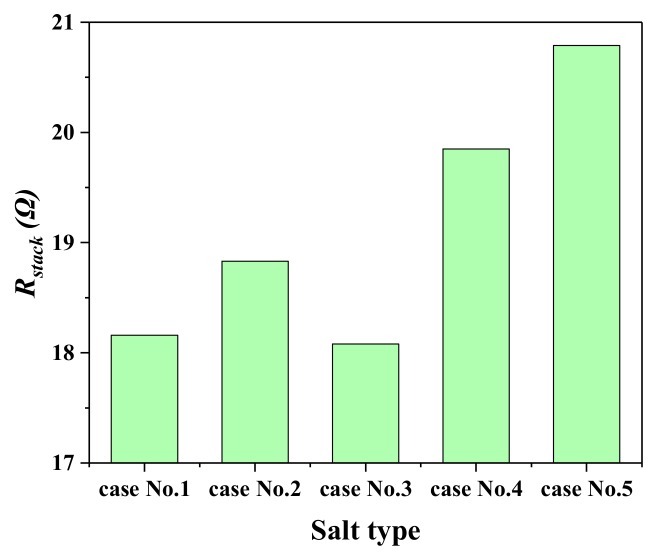
The total stack resistance tested in the open-circuit voltage (OCV) mode, for Case No. 1–5.

The total stack electric resistance was investigated prior to the practical RED operation to survey the fundamental information of the RED stack. The electric resistance was recorded using the electric load under the OCV mode, by maintaining the flow rate at 135 mL·min^−1^ for all the five cases (to avoid the hydrodynamic influence). It is found in Figure 2 that Case No. 3 has the lowest total stack electric resistance (ca. 18 Ω) in the five operations. Case No. 5 has the highest total stack electric resistance (ca. 20.6 Ω), which may be owing to the introduction of the NH_4_^+^ ion. The NH_4_^+^ ion has corresponding different physicochemical characters (bare ion radius (0.148 nm), hydrated radius (0.331 nm), hydration free energy (29.5 kJ/mol-ion)), in comparison to the Na^+^ ion (bare ion radius (0.117 nm), hydrated radius (0.358 nm), hydration free energy (365 kJ/mol-ion)) [20]. They perform absolute different properties when transporting across IEMs.

### 2.2. Polarization Curves of RED

**Figure 3:**
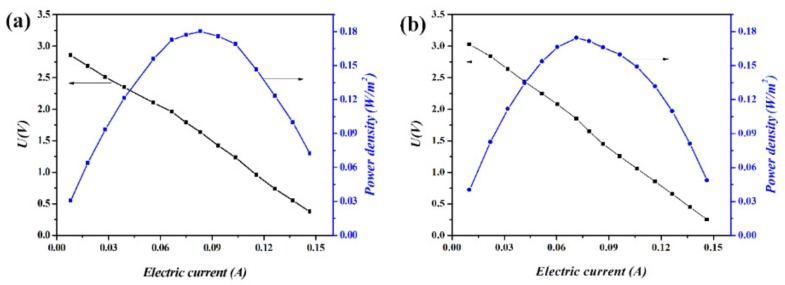
The changes in voltage output (black line) and power density (blue line) in the function of the electric current. (**a**) HCC: 0.5 mol·L^−1^ NaCl; LCC: 0.01 mol·L^−1^ NaCl. (**b**) HCC: 0.49 mol·L^−1^ NaCl + 0.01 mol·L^−1^ NaHCO_3_; LCC: 0.01 mol·L^−1^ NaHCO_3_. The flow rate ratio for HCC and LCC was kept at 180 mL·min^−1^.

The polarization curves for experiments Case No.1 and Case No.3 were investigated for the understanding of the influence of ion composition on power density. When the external load was connected with RED stack, the voltage output U could be calculated as the difference between the electromotive force *E_OCV_* and the voltage drop across the internal resistance R_stack_ (*U* = *E_OCV_* − *I R_stack_*). The changes in voltage output (black line) and power density (blue line) in the function of the electric current was plotted as the two polarization curves, and are shown in Figure 3. It was found that OCV for both operations decreases with the loading current. The maximum *E_OCV_* (OCV) are 2.95 and 3.06 V, while perform the same short-circuit current (0.155 A) for both cases. The maximum power density was 0.180 W ·m^−2^ at the electric current of 0.083 A for Case No.1 (Figure 3a), and 0.176 W·m^−2^ at the electric current of 0.072 A for Case No.3 (Figure 3b). The current when RED reached the maximum outputting power density is lower for Case No.3 in comparison to Case No.1. This may be attributed to the difference in physicochemical properties for species HCO_3_^−^ and Cl^−^, and then reflected as the thermodynamic difference when mixing in the RED stack.

### 2.3. The Discharging Property of RED

By investigating the changes in internal resistance (*R_stack_*), open-circuit voltage (*E_OCV_*), and power density (*P_gross_*), the RED was investigated. This section presents the effects of flow rate and LCC electrolyte composition on the SGP recovery. The flow rates were changed from 45 to 225 mL·min^−1^, with respect to the changes in boundary layer resistance (*R_BL_*).

**Figure 4:**
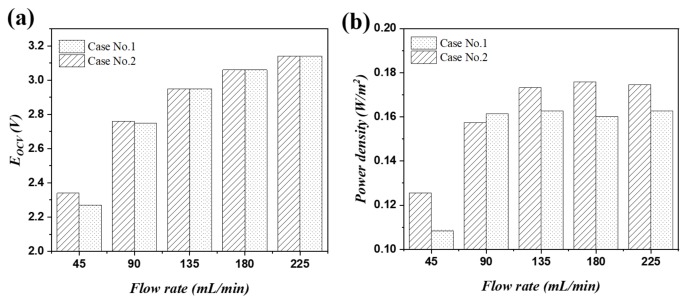
The role of NaCl, NaHCO_3_ as the electrolyte in the LCC and their influence on reverse electrodialysis (RED) transmembrane voltage and power density. (**a**) Open-circuit voltage, (**b**) power density.

To investigate the effect of ion composition in HCC on SGP recovery, 0.5 M NaCl (Case No. 1) in HCC was changed to 0.49 M NaCl + 0.01 M NaHCO_3_ (Case No. 2). Figure 4a shows the effects of flow rate on OCV for Case No.1 and Case No. 2. It was found that the OCV increased from 2.26 to 3.14 V for Case No. 1 when the flow rate was increased from 45 to 225 mL·min^−1^. A similar trend was also found for Case No. 2 (increase from 2.35 to 3.14 V when increasing flow rate from 45 to 225 mL·min^−1^). This phenomenon could be appropriately explained according to the Nernst equation in the multi-component system (Equation 4). The species type and their corresponding activity in the solution, as well as the interaction with the functional group in the membrane matrix co-induced the transmembrane voltage changes for the different electrolyte. Otherwise, the increment on flow rate decreased the boundary layer thickness, and thus mitigated the concentration polarization in the boundary layer, which effectively improves the practical concentration difference on the two sides of the ion exchange membrane. The increment of transmembrane voltage drop by increasing the solution flowing rate could then be explained accordingly. For the power density, it increased from 0.108 to 0.162 W·m^−2^ and 0.125 to 0.176 W·m^−2^ for Case No. 1 and Case No. 2, respectively, by increasing the flow rate from 45 to 225 mL·min^−1^. The increment is significant just by doubling the flow rate to 90 mL·min^−1^, in comparison to the operation at the rate of 135, 180, and 225 mL·min^−1^. The powder density difference when using 0.5 M NaCl|0.01 M NaHCO_3_ and 0.49 M NaCl + 0.01 M NaHCO_3_|0.01 M NaHCO_3_ as the supporting electrolyte in HCC|LCC was mainly attributed to the transition difference in the membrane phase and the concentration distribution in the boundary layer, which was discussed above.

**Figure 5:**
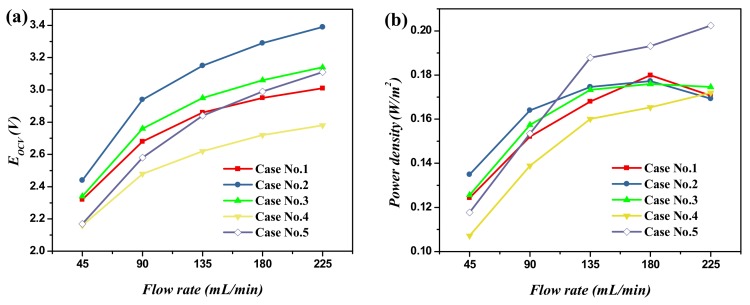
Influence of the concentration ratio of HCC and LCC on RED performance under different flow rates. (**a**) Open-circuit voltage, (**b**) power density. Note. The HCC salt solutions for Case No.1–5 were 0.5 M NaCl, 0.495 M NaCl + 0.005 M NaHCO_3_, 0.49 M NaCl + 0.01 M NaHCO_3_, 0.48 M NaCl + 0.02 M NaHCO_3_, 0.99 M NaCl + 0.01 M NaHCO_3_ solution, respectively.

The concentration difference on the two sides of the membrane is one critical aspect which determines the performance of the RED stack. Therefore, we investigated the process by changing the electrolyte concentration in both the LCC and HCC, and the results are given in Figure 5. Figure 5 demonstrates the changes in *E_OCV_* and the power density of the RED stack under different flow rates. The legends in the figure for five cases are illustrated in Table 2. It is interesting to find OCV increase with the flowing rate for every set of experiments. For example, when using 0.02 M NaHCO_3_ as the electrolyte in the LCC, OCV gradually increases from 2.16 to 2.78 V when increasing the flow rate from 45 to 225 mL·min^−1^. The finding here further confirms the critical role of the concentration polarization effect on the RED, which is not only a general case in natural systems (seawater|river water), but also in complex artificial complex systems. By maintaining all operations under the same flowing rate, it was found that the OCV decreases with the increment of NaHCO_3_ concentration in the LCC. For example, it decreases from 2.44 to 2.16 V by increasing the NaHCO_3_ concentration from 0.005 M NaHCO_3_ to 0.02 M NaHCO_3_ (flow rate of 45 mL·min^−1^).

The power density increases with the flowing rate for every case of the experiments. For example, when using 0.02 M NaHCO_3_ as the supporting electrolyte in the LCC, the power density gradually increases from 0.107 to 0.160 W·m^−2^ by increasing the flow rate from 45 to 135 mL·min^−1^. Additionally, the power density was relatively stable when the flow rate reached 135 mL·min^−1^. By maintaining all the operations under the same flowing rate, it was found that using 0.49 M NaCl + 0.01 M NaHCO_3_|0.01 M NaHCO_3_ as the supporting electrolyte in HCC|LCC (Case No.3) obtained a relatively high and stable power density. The highest power density (0.202 W·m^−2^) was obtained for the system of 0.99 M NaCl + 0.01 M NaHCO_3_|0.01 M NaHCO_3_ (Case No.5). The powder density was mainly determined by two factors, i.e., the OCV and stack resistance. The difference of power density among the five cases was mainly attributed to the ion transition difference in the membrane phase and the concentration distribution in the boundary layer, which was discussed above. When the concentration of NaHCO_3_ was below 0.01 M in LCC, the main influence of power density was the membrane stack internal resistance. In contrast, when the concentration of NaHCO_3_ was over 0.01 M in LCC, the main influence of power density was OCV. Therefore, the RED stack with the feed system of 0.49 M NaCl +0.01 M NaHCO_3_|0.01 M NaHCO_3_ was chosen for further experiments, with high energy utilized efficiency as well as a higher open-circuit voltage.

**Figure 6:**
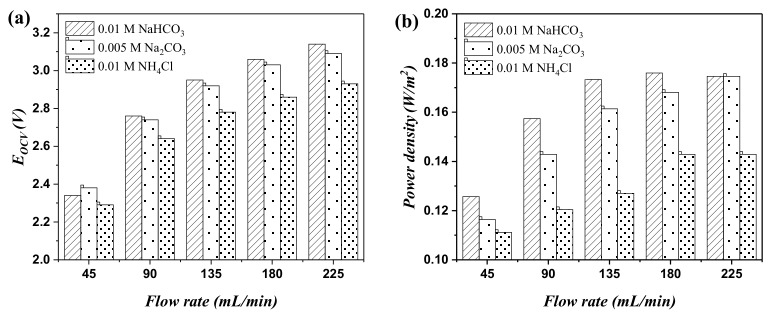
The changes in OCV and power density under different flow rates. (**a**) Open-circuit voltage, (**b**) power density. The legends represent electrolyte kind.

The RED performance was further investigated by changing the electrolyte in the LCC to Na_2_CO_3_ and NH_4_Cl, and HCC to the composites of NaCl + Na_2_CO_3_ and NaCl + NH_4_Cl, respectively. Figure 6a gives the changes in open-circuit voltages on the total RED stack by changing the flow rate. It was found that OCV on RED by using Na_2_CO_3_ as the LCC electrolyte was slightly lower than that using NaHCO_3_ under operating conditions, except for the same flow rate at 45 mL/min. The OCV was further decreased by replacing the electrolyte in LCC to NH_4_Cl. The highest OCV was 3.14, 3.09, and 2.93 V for the NaHCO_3_, Na_2_CO_3_, and NH_4_Cl electrolyte, respectively. The same trend was also found in the power density when changing the flow rates. The power density increases with an increase in the flow rate for all the three electrolytes (the electrolyte composition in LCC). The power density of three investigated electrolytes follows the order: NaHCO_3_ > Na_2_CO_3_ > NH_4_Cl (see Figure 6b). The highest power densities were 0.175, 0.175, and 0.143 W/m^2^ for the NaHCO_3_, Na_2_CO_3_, and NH_4_Cl systems at the 225 mL/min flowing rate, respectively.

## 3. Materials and Methods

### 3.1. Materials

The cation exchange membranes and anion exchange membranes used in the experiments were CJ-MC-3 and CJ-MA-2, respectively (Hefei ChemJoy Polymers Co., Ltd., Hefei, China). The main properties of the ion exchange membranes are listed in Table 3. Before the experiments, the cation and anion exchange membranes were immersed in a 0.5 mol·L^−1^ NaCl solution for 24 h to change them into corresponding Na^+^ and Cl^−^ form. The reagents used in the study, including NaCl, NH_4_Cl, NaHCO_3_, Na_2_CO_3_, K_3_[Fe(CN)_6_], and K_4_[Fe(CN)_6_], were all analytical grade and purchased from Sinopharm Chemical Reagent Co., Ltd., Shanghai, China. Deionized water was used throughout the experiments.

### 3.2. Transmembrane Voltage Test

The transmembrane voltage test was at room temperature using a self-designed experimental setup [21]. The set-up was composed of one membrane and two compartments: HC and LC compartments. The two compartments were separated by the test membrane using a quadrate clip. The two work electrodes were brought close to testing membrane surfaces to record the potential drop across the testing membranes which was recorded by a digital multimeter (VICTOR, VC890C+, VICTOR^®^ YITENSENTM). The effective area of the membranes was 3.8 cm^2^.

### 3.3. RED Experimental Setup

**Figure 7:**
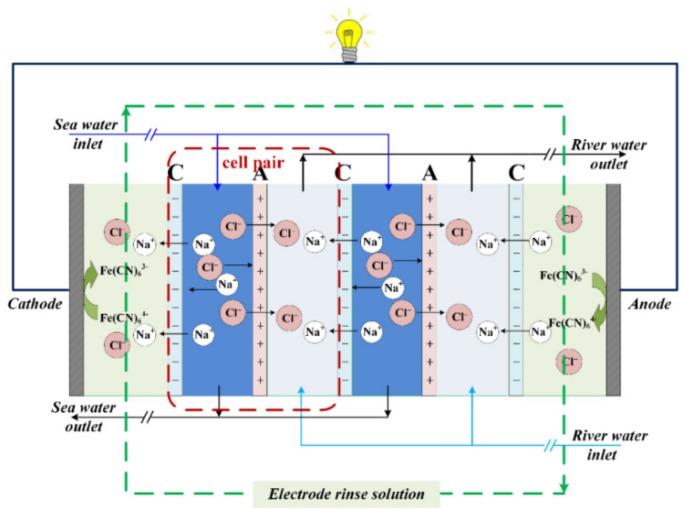
A schematic diagram of the principle of reverse electrodialysis, RED.

The experimental setup of RED was designed and installed in our lab. A schematic diagram of the RED principle is illustrated in Figure 7. The RED setup mainly contains (1) a cathode plate and an anode plate, which was made of titanium coated with ruthenium and iridium with the same effective area and acted as the electron conductor; (2) several cell pairs of cation and anion exchange membranes which were alternately arranged. Twenty repeated cell pairs were used with a total effective area of 20 × 2 × 189 cm^2^ (9 × 21 cm); (3) silica gel spacers with the thickness of 0.75 mm were adopted to separate the anion and cation exchange membrane. In the RED process, three flow streams, i.e., electrode compartment, high salt concentration compartment (HC), and low salt concentration compartment (LC) were established. Electrode rinse solution was circulated with supporting electrolyte using the peristaltic pump (BT600L, Baoding Lead fluid Technology Co., Ltd., Baoding, China) at a flow rate of 90 mL·min^−1^. The same flow rate was maintained in HC and LC with two peristaltic pumps (BT600L, Lead fluid, China). The flow stream was under “feed-and-bleed” mode and circulated through the HC and LC with various velocities (45–225 mL·min^−1^).

### 3.4. RED Performance Tests

A programmable DC electronic load (FT6300A, Shenzhen Faith Technology Co., Ltd., Shenzhen, China) was connected between the anode and cathode to measure the internal resistance, open-circuit voltage (OCV), and the electric current of the RED membrane stack. The stepwise current was set with a scanning rate of 10 mA/s, from 0 A to the maximum current (when the voltage of the membrane stack became reversed). The response curves of voltage vs. electric current and power density vs. electric current were recorded by a computer for each set condition. The electrochemical performance of the RED membrane stack was obtained by evaluating the open-circuit voltage, maximum power density, and maximum current. The open-circuit voltage was determined from the vertical axis intercept of the polarization curves, and the maximum current was obtained from the horizontal axis intercept in the polarization curves.

### 3.5. Determination of the RED System

The line flow velocity (*V*) can be defined as the mean fluid velocity inside a single spacer-filled channel. It can be estimated by Equation (1) [10,22].

(1) $$V=\frac{Q}{N\cdot \delta \cdot b\cdot \varepsilon_{sp}},$$

where *Q* is the volumetric flow rate (mL·min^−1^) in HC or LC inlet, *δ* is the spacer thickness (0.075 cm), *b* is the compartment width (9 cm for the small stack), and *ε_sp_* is the spacer porosity (75% for the woven spacer used in this study). For simplicity, the flow rate in the experiment is expressed as the volumetric flow rate.

The power of the RED stack was calculated using Equation (2)

(2) P = UI

where P is the power of the membrane stack (W), U is the voltage of the membrane stack (V), I is the scanned current (A).

To test the performance of RED, LC and HC were fed with solutions of 0.01 and 0.5 mol·L^−1^ NaCl, respectively. Electrode rinse solution consisted of 0.05 M K_3_ [Fe(CN)_6_], 0.05 M K_4_[Fe(CN)_6_]·3H_2_O and 0.25M NaCl, and was circulated in the electrode compartment at the flow rate of 90 mL·min^−1^.

To investigate the influence of ion composition on the RED performance, the HC was also fed with 0.5 mol·L^−1^ NaCl and 0.49 mol·L^−1^ NaCl + 0.01 mol·L^−1^ NaHCO_3_, while the LC was 0.01 mol·L^−1^ NaHCO_3_. Otherwise, the LC solution concentration was changed (0.005 to 0.02 M NaHCO_3_), while fixing the HC solution as 0.495 mol·L^−1^ NaCl + 0.0051 mol·L^−1^ NaHCO_3_ and 0.48 mol·L^−1^ NaCl + 0.02 M NaHCO_3_, to test the influence of LC solution. The HC was also fed with 0.99 mol·L^−1^ NaCl + 0.01 mol·L^−1^ NaHCO_3_, while the LC was 0.01 mol·L^−1^ NaHCO_3_, to test the influence of HC solution concentration.

## 4. Theory

The theoretical Nernst potential over one cell ion exchange membrane for sodium chloride single medium system was calculated using the Nernst equation in Equation (3) [23,24].

(3) $$E_{\mathrm{cell}}=\alpha_{CEM}\frac{RT}{F}\ln(\frac{\gamma_{c}^{Na^{+}}\cdot C_{c}^{Na^{+}}}{\gamma_{d}^{Na^{+}}\cdot C_{d}^{Na^{+}}})+\alpha_{AEM}\frac{RT}{F}\ln(\frac{\gamma_{c}^{Cl^{-}}\cdot C_{c}^{Cl^{-}}}{\gamma_{d}^{Cl^{-}}\cdot C_{d}^{Cl^{-}}}).$$

For sodium chloride and sodium bicarbonate double medium system, considering the two kinds anions of Cl^−^ and HCO_3_^−^ migration through anion exchange membrane, as well as referring to the multi-valent ions’ climbing description in a RED process, the multi-ionic expression of the Nernst equation was revised as below.

(4) $$E_{\mathrm{cell}}=\alpha_{CEM}\frac{RT}{F}\ln(\frac{\gamma_{c}^{Na^{+}}\cdot C_{c}^{Na^{+}}}{\gamma_{d}^{Na^{+}}\cdot C_{d}^{Na^{+}}})+\alpha_{AEM}\frac{RT}{F}[\ln(\frac{\gamma_{c}^{Cl^{-}}\cdot C_{c}^{Cl^{-}}}{\gamma_{d}^{Cl^{-}}\cdot C_{d}^{Cl^{-}}})+\ln(\frac{\gamma_{c}^{HCO_{3}{}^{-}}\cdot C_{c}^{HCO_{3}{}^{-}}}{\gamma_{d}^{HCO_{3}{}^{-}}\cdot C_{d}^{HCO_{3}{}^{-}}})].$$

Then, the theoretical open circuit potential of the whole membrane stack was simplified into Equation (5).

(5) $$E_{OCV}=N\frac{(\alpha_{CEM}+\alpha_{AEM})RT}{zF}\ln(\frac{a_{c}}{a_{d}}).$$

The power produced is determined by the electrochemical potential drop across the membrane stack (*E_OCV_*), the stack resistance, and external load resistance (*R_load_*) resulting in Equation (6) [12,25].

(6) $$P=I^{2}R_{load}=\frac{E_{OCV}{}^{2}}{{\left(R_{stack}+R_{load}\right)}^{2}}R_{load}.$$

The maximum power output of the RED system is obtained when *R_load_* equals the resistance of the stack (*R_stack_*). Thus, the maximum power output can be simplified into Equation (7).

(7) $$P_{max}=\frac{E_{OCV}{}^{2}}{4R_{stack}}.$$

Consequently, the gross power density (power output per unit membrane area, *P_gross_*) was calculated from *P_max_*, which is shown in Equation (8) [10].

(8) $$P_{gross}=\frac{P_{max}}{2AN}=\frac{E_{OCV}{}^{2}}{8ANR_{stack}},$$

where *P_gross_* is the maximum gross power density (W·m^−2^), *P_max_* is maximum power output (W), *A* is effective area of a single ion exchange membrane (m^2^).

The total electric resistance of the RED stack includes the parts of electrodes, electrolytes, membranes, and diffusion boundary layers on the membrane-solution interface. Simplified models neglect the resistance of diffusion boundary layers and combine its contribution with membrane resistance and express the overall resistance (Ω) as Equation (9) [10].

(9) $$R_{stack}=\frac{N}{A}(R_{a}+R_{c}+\frac{d_{c}}{\kappa_{c}}+\frac{d_{d}}{\kappa_{d}})+R_{el},$$

where *A* is a single effective membrane area (cm^2^); *R_a_* is the area resistance (Ω·cm^2^) of anion exchange membrane; *R_c_* is the area resistance (Ω·cm^2^) of cation exchange membrane; *R_el_* is the resistance (Ω) of electrodes; *d_c_* is the thickness (cm) of HC; *d_d_* is the thickness of LC; *κ_c_* is the specific conductivity (mS·cm^−1^) of the concentrated solution; and *κ_d_* is the specific conductivity (mS·cm^−^^1^) of the diluted solution.

The electrode resistance was negligible when the repeated membrane pairs were more substantial than 20 pcs. Then the entire RED stack resistance was expressed as Equation (10) [26,27].

(10) $$R_{stack}=\frac{N}{A}(R_{ohmic}+R_{\Delta C}+R_{BL}),$$

where *R_ohmic_* is the membrane resistance ascribed to the ionic transport through the membranes, which is equal to the one cell resistance discussed above. *R_△C_* is the resistance ascribed to the reduced electromotive forces as a consequence of the change in the concentration of the bulk solution. It considers the change of the solution concentration from the inlet to the outlet of the solution compartment with the spatial difference of membrane potential (*y*-axis). *R_BL_* is the boundary layer resistance due to concentration polarization which is induced by diffusive boundary layer near the membranes at lower flow rates (*x*-axis). Both *R_△C_* and *R_BL_* are kinds of non-ohmic resistance.

The net power density (*P_net_*, in W·cm^−^^2^) that generates in a RED stack is the difference between the gross power density and the power consumed on solution pumping (pressure drop over the inlet and outlet channel). Thus, the net power density can be expressed as Equation (11) [10].

(11) $$P_{net}=P_{gross}-P_{pump}=\frac{E_{OCV}{}^{2}}{8ANR_{stack}}-\frac{\Delta p_{c}Q_{c}+\Delta p_{d}Q_{d}}{NA},$$

where ∆*p_c_* and ∆*p_d_* are the pressure drops along HC and LC, respectively. *Q_c_* and *Q_d_* are the volumetric flow rates of HC and LC, respectively.

## 5. Conclusions

This work investigated the influence of ion composition on the trans-membrane potential across the ion exchange membrane (IEM), for a better understanding of “reverse electrodialysis heat engine” when running in the complex ion composition. The artificially prepared solutions NaHCO_3_, Na_2_CO_3_, and NH_4_Cl circulate inside the RED stack, as the alternative of the conventional NaCl systems. The evaluative criteria of the RED performance, i.e., open-circuit voltage, gross power density, and short-circuit current were introduced for the evaluation of the process. It was found that the new system (0.49 M NaCl + 0.01 M NaHCO_3_|0.01 M NaHCO_3_) output a relatively stable power density (0.174 W·m^−2^), with the open-circuit voltage 2.95 V under the low flow rate of 0.22 cm/s. Meanwhile, the simulated natural system (0.5 M NaCl|0.01 M NaCl) output the power density 0.168 W·m^−2^, with the open-circuit voltage 2.86 V under the low flow rate of 0.22 cm/s. The work will advance the understanding of reverse electrodialysis heat engine process, as well as the performance of RED when running in complex systems (wastewater).

## Figures and Tables

**Table 1:** The ion composition in the high salt concentration compartment (HCC) and low salt concentration compartment (LCC) in the experiments.

Salt Type	HCC	LCC
Case No.1	0.5 M NaCl	0.01 M NaCl
Case No.2	0.5 M NaCl	0.01 M NaHCO_3_
Case No.3	0.49 M NaCl + 0.01 M NaHCO_3_	0.01 M NaHCO_3_
Case No.4	0.49 M NaCl +0.005 M Na_2_CO_3_	0.005 M Na_2_CO_3_
Case No.5	0.49 M NaCl + 0.01 M NH_4_Cl	0.01 M NH_4_Cl

**Table 2:** The ion composition in the HCC and LCC in the experiments.

Salt Type	HCC	LCC
Case No.1	0.5 M NaCl	0.01 M NaCl
Case No.2	0.495 M NaCl + 0.005 M NaHCO_3_	0.005 M NaHCO_3_
Case No.3	0.49 M NaCl + 0.01 M NaHCO_3_	0.01 M NaHCO_3_
Case No.4	0.48 M NaCl +0.02 M NaHCO_3_	0.02 M NaHCO_3_
Case No.5	0.99 M NaCl + 0.01 M NaHCO_3_	0.01 M NaHCO_3_

**Table 3:** The main characteristics of the membranes used in the experiments.

Properties	CJ-MC-3	CJ-MA-2
Thickness/mm	0.220	0.185
Ion exchange capacity/mmol/g	1.50	1.25
Water Uptake/%	35	32
Resistance/Ω·cm^2^	3.0	1.5
Transfer number/%	98	99
Break stress/MPa	>3.5	>3.5

## Abbreviations

*A*	the effective area of a single membrane (m^2^)
*a*_*c*_	the activity of the concentrated salt solution (mol·L^−1^)
*a*_*d*_	the activity of the diluted salt solution (mol·L^−1^)
*AEM*	anion exchange membrane
*b*	compartment width (cm)
*C*_*c*_	the concentration of HC (mol·L^−1^)
*C*_*d*_	the concentration of LC (mol·L^−1^)
*CEM*	cation exchange membrane
*d*_*c*_	the thickness of HCC (cm)
*d*_*d*_	the thickness of LCC (cm)
*F*	Faraday constant (96485 C·mol^−1^)
*HCC*	high salt concentration compartment
*I*	current (A)
*LCC*	low salt concentration compartment
*N*	number of cell pairs for RED
*OCV*	open circuit voltage (V)
*P*	output power (W)
*P*_*gross*_	maximum gross power density (W·m^−2^)
*P*_*max*_	maximum output power (W)
*P*_*net*_	net power density (W·m^−2^)
*P*_*pump*_	power density consumed on pumping (W·m^−2^)
*Q*	volumetric flow rate (mL·min^−1^)
*Q*_*c*_	the volumetric flow rate of HC (L·s^−1^)
*Q*_*d*_	volumetric flow rate of LC (L s^−1^)
*R*	gas constant (8.314 J·mol^−1^·K^−1^)
*R*_*a*_	the area resistance of anion exchange membrane (Ω·m^2^)
*R*_*BL*_	boundary layer resistance (Ω)
*R*_*c*_	the area resistance of cation exchange membrane (Ω·m^2^)
*R*_*el*_	the resistance of electrodes (Ω)
*R*_*load*_	load resistance (Ω)
*R*_*ohmic*_	ohmic resistance (Ω)
*R*_*stack*_	stack resistance (Ω)
*R*_*△C*_	concentration difference resistance (Ω)
*RED*	reverse electrodialysis
*SGP*	salinity gradient power
*T*	absolute temperature (K)
*U*	voltage output
*V*	line flow velocity (cm·min^−1^)
*z*	electrochemical valence
*α*_*AEM*_	permselectivity of the anion exchange membrane
*α*_*CEM*_ **	permselectivity of the cation exchange membrane
*δ*	spacer thickness (cm)
*ε*_*sp*_	spacer porosity
*κ*_*c*_	the specific conductivity of the concentrated solution (mS·cm^−1^)
*κ*_*d*_	the specific conductivity of the diluted solution (mS·cm^−1^)
∆*p*_*c*_	the pressure drops along HC (KPa)
∆*p*_*d*_	the pressure drops along LC (KPa)
